# Evolving Roles of Muscle-Resident Fibro-Adipogenic Progenitors in Health, Regeneration, Neuromuscular Disorders, and Aging

**DOI:** 10.3389/fphys.2021.673404

**Published:** 2021-04-20

**Authors:** Marine Theret, Fabio M. V. Rossi, Osvaldo Contreras

**Affiliations:** ^1^Biomedical Research Centre, Department of Medical Genetics, School of Biomedical Engineering, The University of British Columbia, Vancouver, BC, Canada; ^2^Departamento de Biología Celular y Molecular, Center for Aging and Regeneration (CARE-ChileUC), Facultad de Ciencias Biológicas, Pontificia Universidad Católica de Chile, Santiago, Chile; ^3^St. Vincent’s Clinical School, Faculty of Medicine, UNSW Sydney, Kensington, NSW, Australia; ^4^Developmental and Stem Cell Biology Division, Victor Chang Cardiac Research Institute, Darlinghurst, NSW, Australia

**Keywords:** skeletal muscle fibrosis, muscle FAPs, muscle regeneration, aging, muscle stem cells (MuSCs), macrophages, extracellular matrix (ECM), duchenne muscular dystrophy (DMD)

## Abstract

Normal skeletal muscle functions are affected following trauma, chronic diseases, inherited neuromuscular disorders, aging, and cachexia, hampering the daily activities and quality of life of the affected patients. The maladaptive accumulation of fibrous intramuscular connective tissue and fat are hallmarks of multiple pathologies where chronic damage and inflammation are not resolved, leading to progressive muscle replacement and tissue degeneration. Muscle-resident fibro-adipogenic progenitors are adaptable stromal cells with multilineage potential. They are required for muscle homeostasis, neuromuscular integrity, and tissue regeneration. Fibro-adipogenic progenitors actively regulate and shape the extracellular matrix and exert immunomodulatory functions via cross-talk with multiple other residents and non-resident muscle cells. Remarkably, cumulative evidence shows that a significant proportion of activated fibroblasts, adipocytes, and bone-cartilage cells, found after muscle trauma and disease, descend from these enigmatic interstitial progenitors. Despite the profound impact of muscle disease on human health, the fibrous, fatty, and ectopic bone tissues’ origins are poorly understood. Here, we review the current knowledge of fibro-adipogenic progenitor function on muscle homeostatic integrity, regeneration, repair, and aging. We also discuss how scar-forming pathologies and disorders lead to dysregulations in their behavior and plasticity and how these stromal cells can control the onset and severity of muscle loss in disease. We finally explore the rationale of improving muscle regeneration by understanding and modulating fibro-adipogenic progenitors’ fate and behavior.

## Introduction

Although reduced muscle function secondary to trauma, disease, neuromuscular disorders, and age-related sarcopenia affects millions of people each year, little is known about the origins of ectopic muscle scarring and the molecular pathways that underlie its development. Adult skeletal muscle is known not only for its ability to expand following hypertrophic stimuli or shrink when disused but also to restore its mass and function after injury. In this regenerative process, a key event is the presence of muscle-resident stem cells, a tightly coordinated immune response, and the transient deposition of a supportive matrix that eventually is reabsorbed when regeneration is successful. Thus, several precisely timed events need to take place for successful muscle regeneration requiring numerous cell types to engage in a complex network of cellular interactions through autocrine, paracrine, and juxtacrine signaling (Lemos et al., 2015; Malecova et al., 2018; Scott et al., 2019; Chazaud, 2020; Oprescu et al., 2020).

In the past decade, we have learned that efficient skeletal muscle regeneration does not rest only on muscle stem cells (MuSCs). Indeed, many different cell types -resident and non-resident- contribute to the cellular composition of muscles, including pericytes, endothelial cells, smooth muscle cells, fibro-adipogenic progenitors (FAPs), various immune cells, tenocytes, and nerve-associated cells such as glia and Schwann cells, all of which participate in this complex biological process (Blau et al., 2015; Mashinchian et al., 2018; Wosczyna and Rando, 2018). These diverse and heterogeneous cells act together to maintain muscle functions, tissue integrity, and regenerative properties.

Even though muscles are highly regenerative following acute damage, chronic pathologies often cause an increased and dysregulated accumulation of connective tissue (CT) and fat – also known as fibro-fatty infiltration- especially in muscle degenerative conditions associated with persistent or chronic inflammation (Grounds, 2008; Mann et al., 2011). Thus, fibro-fatty infiltration is commonly observed in several myopathies and neuromuscular disorders and correlates with the pathology’s progression and extension. Muscle fibro-fatty scarring is also present after repeated cycles of damage, denervation, amyotrophic lateral sclerosis (ALS), rotator cuff tears, and during aging-related sarcopenia (discussed below in detail). Albeit to a different extent in every situation.

Continued deposition and increased stiffening of the matrix usually support a biochemical, biophysical, and biomechanical pro-fibrotic feedback loop sustaining stromal cell persistence in the tissue beyond their normal kinetics (Hinz and Lagares, 2020). This self-perpetuating mechanism is a well-known cellular and pathological hallmark of detrimental tissue degeneration and fibrosis. Therefore, changes in matrix quantity and quality, increased matrix complexity and tissue stiffness may help tip the affected tissue into a self-perpetuating pathological state. In this abnormal setting, the bioavailability of damage-induced cues is magnified and may lead to scarring in response to insults that would generally be below the threshold required (Klingberg et al., 2014; Karsdal et al., 2017; Murray et al., 2017; Pakshir et al., 2020). Although significant progress in understanding these phenomena has been made at the tissue level, a big question in the muscle field has been identifying and characterizing the cell(s) that produce ectopic fibrous, fat, and bone in degenerated muscles.

Muscle-resident, PDGFRα-expressing FAPs have emerged as crucial players in tissue homeostasis, regeneration, and disease. These stromal cells expand clonally and have the ability to differentiate into stromal cell lineages, including fibroblast, adipocyte, chondrocyte, and osteocyte. In their multipotent state, FAPs modulate many signaling pathways. They produce crucial growth factors like IGF-1, TGF-β, Follistatin, CTGF/CCN2, WNTs, PDGFs, and multiple ILs (for a recent review, see Biferali et al., 2019). They also produce a plethora of matrix or matrisome proteins like collagens, proteoglycans (e.g., Decorin), integrins, laminins, and fibronectin, which impact the cellular physiology of many resident cells and non-resident cells in homeostasis, upon injury and disease.

Here, we outline the current knowledge of FAPs contributions to muscle homeostasis, neuromuscular integrity, regeneration, repair, and aging. We also discuss how their fate is regulated in a context-dependent manner and how the tissue microenvironment largely dictates FAP plasticity and behavior. Finally, we highlight therapeutic opportunities stemming from targeting these precursor cells and their activities to stimulate muscle regeneration in neuromuscular disorders, pathology, and aging.

## Muscle Growth, Long-Term Maintenance, and Regeneration Require Unipotent Muscle Stem Cells

Skeletal muscle has a particular capacity to regenerate fully (*restitutio ad integrum*) even after several rounds of injury (Günther et al., 2013; Tedesco et al., 2018). This remarkable ability is mainly attributable to a reservoir of quiescent adult MuSCs, called satellite cells (reviewed in Wang and Rudnicki, 2012; Almada and Wagers, 2016). In brief, these non-interstitial adult stem cells localize in between the muscle sarcolemma and the myofibers’ basal lamina (Katz, 1961; Mauro, 1961; Church et al., 1966). Aiming to efficiently rebuild muscle following injury, MuSCs exit quiescence, activate, proliferate, differentiate, and fuse either with one another to form multinucleated muscle cells -myofibers- or with pre-existent damaged myofibers (Kuang et al., 2006; Robertson et al., 1990; Rudnicki et al., 2008; Sacco et al., 2008; Von Maltzahn et al., 2013). As functional adult unipotent muscle-resident stem cells, MuSCs can also self-renew to maintain the stem cell pool (reviewed in Relaix and Zammit, 2012). Genetic lineage-ablation of Pax7+ MuSCs demonstrated that muscle regeneration requires these adult stem cells (Lepper et al., 2011; Murphy et al., 2011; Sambasivan et al., 2011; Fry et al., 2015). Indeed, the absence of MuSCs leads to severe fibro-fatty deposition after myotrauma, resulting from the inability to rebuild the lost tissue (Lepper et al., 2011; Murphy et al., 2011; Sambasivan et al., 2011). Prepubertal skeletal muscle growth also requires MuSCs (Bachman et al., 2018), which once again demonstrates the essential functions of muscle stem cells in adult muscle regeneration and growth. Remarkably, the expression of Pax7 itself is also crucial for muscle regeneration since its gene deletion impairs MuSC self-renewal and causes loss of regeneration following single or several rounds of injury (Oustanina et al., 2004; Günther et al., 2013; Von Maltzahn et al., 2013).

A growing body of work demonstrated that MuSCs are chronically activated during muscle pathology, which leads to their exhaustion and impaired skeletal muscle regeneration (Heslop et al., 2000; Sacco et al., 2010; Dadgar et al., 2014). This phenomenon also occurs in several neuromuscular disorders and contributes to disease progression and poor regenerative outcomes (Castets et al., 2011; Kudryashova et al., 2012; Ross et al., 2012). Hence, diseases marked by chronic muscle degeneration cause dysregulated MuSC fate and behavior. How pathology-driven satellite cell depletion could affect the pool of stromal cells (e.g., FAPs) is unknown, and therefore, further research is needed to address this question.

## Fibro-Adipogenic Progenitors Are Essential for Efficient and Sufficient Skeletal Muscle Regeneration and Required for Homeostatic Neuromuscular Integrity

Initial observations suggested that muscle-resident TCF7L2 (also known as T-cell factor 4, TCF4) expressing stromal cells regulate normal muscle development (Kardon et al., 2003; Mathew et al., 2011). Later, Vallecillo-García et al. (2017) showed that embryonic OSR1+ mesenchymal progenitor cells significantly contribute to adult fibro-adipogenic progenitors. The authors also reported that these muscle connective tissue cells regulate embryonic myogenesis. Remarkably, both cell lineages, TCF7L2 and OSR1 expressing cells, overlap during embryonic limb development (Vallecillo-García et al., 2017). In the adult, stromal TCF7L2+ cells and OSR1+ cells expand and accumulate after injury and disease (Contreras et al., 2016, 2020; Stumm et al., 2018). These include acute glycerol injury (Contreras et al., 2020), denervated muscles (Contreras et al., 2016), dystrophic muscles of the *mdx* mice (Acuña et al., 2014; Contreras et al., 2020), chronic chemical damage with BaCl_2_ (Contreras et al., 2016), human skeletal muscle injury (Mackey et al., 2017), and in murine muscles of the symptomatic ALS transgenic mice hSOD1G93A (Gonzalez et al., 2017).

Aiming to understand the contribution of TCF7L2 expressing cells to muscle regeneration, Murphy et al. (2011) showed that the genetic lineage-ablation of about 40% of TCF72+ cells caused premature satellite cell differentiation and exhaustion of their regenerating pool, leading to a reduced size of the regenerated myofibers. The authors suggested that efficient muscle regeneration requires the interaction between TCF7L2+ stromal cells and satellite cells. In humans, interstitial TCF7L2 expressing cells also play a supportive role in myogenesis and skeletal muscle regeneration (Mackey et al., 2017). We recently showed that the isolation through a pre-plating strategy of highly adherent muscle connective tissue fibroblasts allows the culture of a high proportion (∼90%) of PDGFRα expressing FAPs (Contreras et al., 2019b). These isolated stromal cells also express the Wnt-responsive TCF7L2 transcription factor (Contreras et al., 2020). These findings strongly suggested a role for stromal cells in regulating skeletal muscle health and regeneration.

**FIGURE 1:**
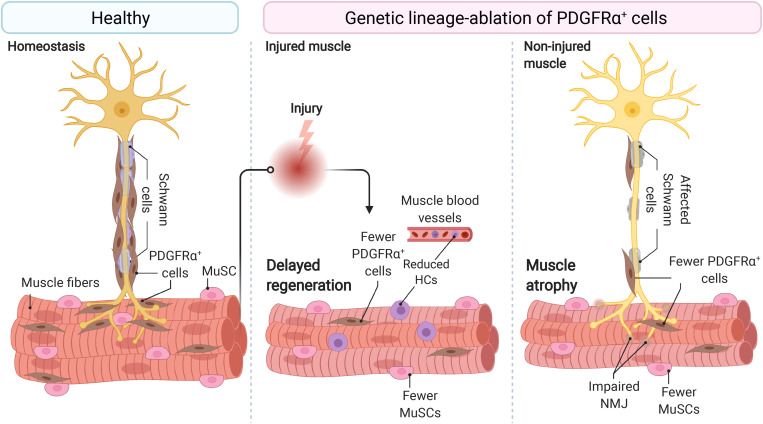
Fibro-adipogenic progenitors are essential and required for efficient skeletal muscle regeneration and homeostatic neuromuscular integrity. Skeletal muscle FAPs are abundant stromal cells, reside within the connective tissue, and lie near the nerve parenchyma and NMJs in healthy muscles and homeostatic conditions. Conditional ablation of FAP+ cells using diphtheria toxin resulted in delayed regeneration and reduced numbers of MuSCs and HCs. In homeostasis and non-damaged muscles, widespread ablation of FAPs causes muscle atrophy, impair Schwann cell, and MuSC behavior and abundance. Impaired NMJ architecture and partial and complete muscle denervation also result from FAP cell ablation without damage. MuSC, muscle stem cell; HCs, hematopoietic cells; NMJ, neuromuscular junction. Motoneuron and Schwann cells are not shown in the middle panel because of space constraints and improved readability.

Roberts et al. (2013) reported that muscle-resident stromal cells expressing Fibroblast Activation Protein alpha (also known as FAPα) also express CD140a (PDGFRα), SCA-1, and CD90. The later three proteins are known markers of murine FAPs (Petrilli et al., 2020). The authors also showed that depletion of Fibroblast Activation Protein alpha expressing cells causes rapid weight loss, reduced skeletal muscle mass, and muscle atrophy (Roberts et al., 2013) (Figure 1). These changes are associated with increased expression of known atrophy-related genes, including *Atrogin-1* and *MuRF,1* and decreased expression of *Follistatin* and the Laminin gene *Lama2*, explaining the muscle atrophy. In response to depletion of stromal cells that express Fibroblast Activation Protein alpha, this cachexic phenotype was also accompanied by altered hematopoiesis. Remarkably, cachectic mice bearing the C26 colon carcinoma showed a reduced number of Fibroblast Activation Protein alpha+ cells in hindlimb muscles, which again suggests the supportive role of these stromal cells in maintaining muscle mass and integrity (Roberts et al., 2013). Hence, this key and often forgotten study suggests that stromal cells (likely FAPs) are indispensable and necessary for skeletal muscle homeostasis and maintenance.

To better understand whether FAPs are necessary for normal skeletal muscle regeneration and homeostatic maintenance, Wosczyna et al. (2019) ablated PDGFRα+ cells using a diphtheria toxin mouse model. The lack of PDGFRα+ cells and their lineage resulted in a decreased MuSC number during muscle regeneration and consequently, smaller myofibers. CD45+ cell number also declined in FAP-depleted muscles at day 3 post-injury. The authors utilizing regeneration assays, isotopic transplants, and FAP transplantation experiments elegantly demonstrated that FAPs are necessary for normal skeletal muscle regeneration. The authors also showed that FAP-ablated mice had about 30–40% less lean mass and generated significantly less force at 3, 6, and 9 months than non-ablated mice (Wosczyna et al., 2019) (Figure 1). These results demonstrated that PDGFRα+ FAPs are required for long-term homeostatic integrity and growth of skeletal muscle. Hence, the depletion of FAPs resulted in muscle regenerative deficits upon injury and atrophy in undamaged conditions.

These results were recently confirmed by the Tsuchida group, who demonstrated that the specific depletion of PDGFRα+ expressing progenitors caused bodyweight reduction and decreased muscle strength and weight (Uezumi et al., 2021). Importantly, these changes were not attributable to decreased food intake in FAP-ablated mice. Although the number of myofibers was unchanged, the authors described reduced myofiber cross-sectional area and increased expression of the muscle-specific E3 ubiquitin ligase *MAFbx*, also known as *Atrogin-1*, in the ablated-muscles without overt signs of muscle injury or inflammation. Notably, PDGFRα+ cell transplantation into the ablated mice’s *tibialis anterior* muscle recovered muscle mass and fiber size (Uezumi et al., 2021). Hence, three groups independently have demonstrated that muscle-resident PDGFRα+ FAPs are indispensable for steady-state muscle maintenance and integrity (Roberts et al., 2013; Wosczyna et al., 2019; Uezumi et al., 2021) (Figure 1).

Muscle wasting associates with changes in myofiber type and muscle-nerve communication defects. Uezumi et al. (2021) also observed that PDGFRα+ cells lie adjacent to motor nerve axons, Schwann cells, and cover the neuromuscular junction (NMJ) in undamaged muscles. In line with this, the genetic ablation, by Cre-mediated expression of diphtheria toxin, of PDGFRα+ FAPs reduced the number of innervated NMJs and increased the proportion of partially denervated and denervated NMJs after 17 days of tamoxifen treatment (Figure 1). These effects increase at longer times (Uezumi et al., 2021). Remarkably, Schwann cell organization and gene expression were also disrupted by depletion of PDGFRα+ FAPs. Mechanistically, the authors suggested that FAP-derived BMP3B is functionally relevant to maintaining muscle mass and integrity (Uezumi et al., 2021). These results collectively suggest that PDGFRα FAPs are required and sufficient for steady-state maintenance of the neuromuscular synapse and nerve-muscle communication and function.

### The Supportive Role of Muscle-Resident Fibro-Adipogenic Progenitors on Myogenesis

**FIGURE 2:**
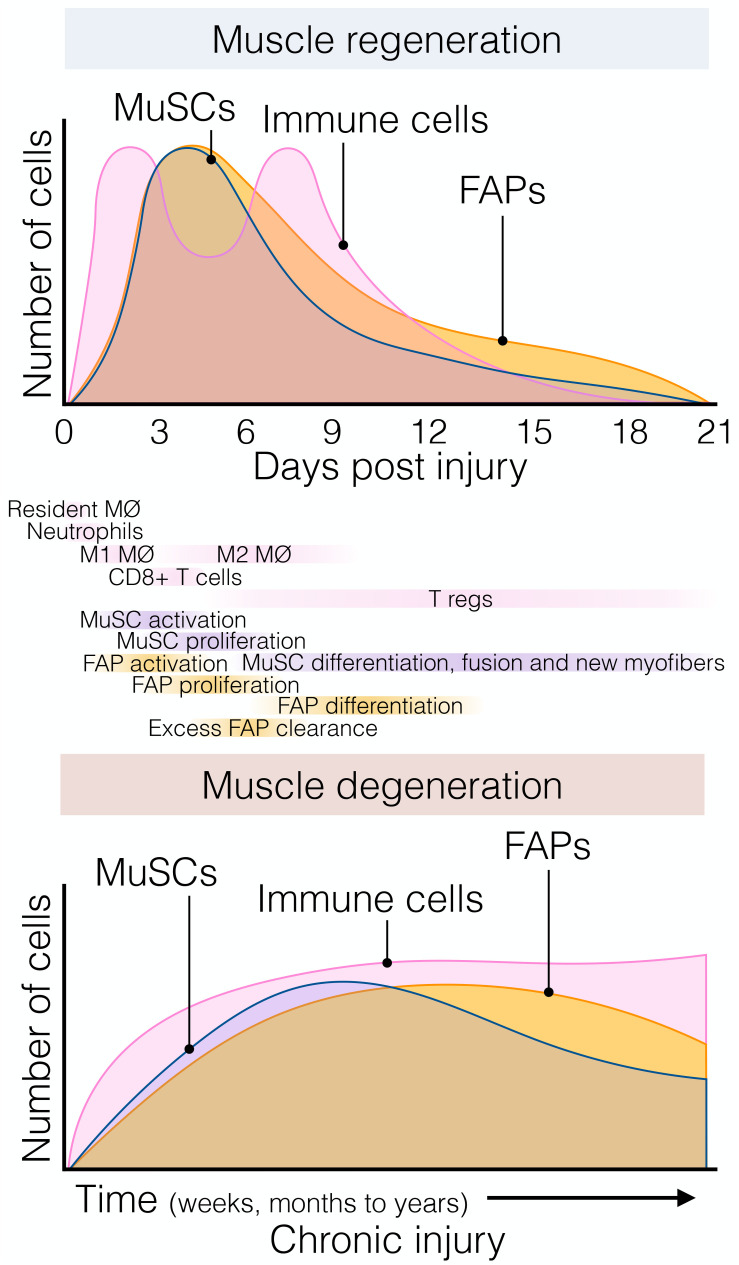
The dynamic flux of muscle stromal, satellite and immune cells cellular flux responds to acute and chronic injury. **(Top)** Muscle regeneration is orchestrated by a synchronous array and flux of several muscle-resident and non-resident cells that interplay to maintain muscle structural integrity and function following injury. Followed the quick and early immune response of resident and non-resident macrophages, neutrophils, and eosinophils; FAPs and MuSCs rapidly activate and proliferate around day 3–5 post-damage. Here, FAPs provide constant support to myogenesis and actively participate in matrix remodeling. This process occurs in parallel with macrophage skewing, and the presence of regulatory Ts-cells follows it. Later, their lineage cells start committing and differentiating. By around day 21 post-injury, injured muscles have regenerated, and damage-induced cellular flux returned to steady-state levels. **(Bottom)** Chronic muscle injury is characterized by progressive muscle degeneration and a dysregulated burst of immune, MuSC, and FAP cells, among other cell types (not shown here for simplification purposes). Several neuromuscular disorders and muscle diseases are often characterized by chronic cycles of muscle degeneration and regeneration. However, there is less knowledge and characterizations about these cellular compartments’ behavior compared to single rounds of injury. Aging and prolonged disease states cause MuSC exhaustion, and FAPs seem to respond similarly. To date, Duchenne muscular dystrophy (DMD) represents the better-characterized muscle disease, but it is unknown whether this gained knowledge can be applied to other disease settings. Tregs, muscle regulatory T-cell; FAP, fibro-adipogenic progenitor; MuSC, muscle stem cell; MØ, macrophage.

In response to injury, skeletal muscle displays a dynamic multicellular response that involves several cell types and discrete regenerative steps (Figure 2, top) (Bentzinger et al., 2013; Chazaud, 2020). The initial and rapid inflammatory response is followed by the concomitant activation of two quiescent muscle-resident cells, MuSCs, and interstitial PDGFRα+ FAPs. Both populations reside close to each other but are separated by the myofiber-associated basal lamina. FAPs and MuSCs proliferate at enormous rates following injury, progressively increasing their numbers from days 2 to 5. From this time until 14–21 days after injury, when damage is resolved, they steadily return to their basal numbers (Figure 2, top) (Joe et al., 2010; Uezumi et al., 2010; Murphy et al., 2011; Lemos et al., 2015; Kopinke et al., 2017; Contreras et al., 2019a; Scott et al., 2019). Remarkably, although this crucial stromal-muscle stem cell interaction was suggested to participate in skeletal muscle regeneration a long time ago (Church, 1970), only recently cumulative evidence have demonstrated that FAPs regulate MuSC fate and behavior, and vice versa (Joe et al., 2010; Uezumi et al., 2010, 2021; Mathew et al., 2011; Murphy et al., 2011; Fiore et al., 2016; Moratal et al., 2019; Wosczyna et al., 2019). In this context, FAPs are principally immunomodulatory cells secreting a large number of factors (discussed below) (Heredia et al., 2013; Scott et al., 2019; Uezumi et al., 2021). FAPs are also responsible for the muscle ECM remodeling after damage, producing a transient ECM (Scott et al., 2019) and supporting MuSC expansion, differentiation, and self-renewal (Figure 2, top) (Heredia et al., 2013; Mozzetta et al., 2013). In addition, it has been shown that the muscle ECM component Collagen VI, secreted from FAPs, specifically regulates MuSC quiescence (Urciuolo et al., 2013). Thus, the correct regulation of basal membrane and ECM composition by FAPs is critical for normal muscle regeneration. Indeed, the biophysical and biomechanical properties of the ECM vary depending on its composition, and impact the fate of MuSCs (Lutolf and Blau, 2009). Gilbert et al. (2010), used bioengineered substrates of varying stiffness to recapitulate key biomechanical characteristics of the MuSCs niche. The authors showed that increased elastic modulus 10^6^ kPa (rigid plastic dishes) stimulates MuSC motility, but 12 kPa (mimicking the elasticity of muscle) promotes MuSC proliferation and muscle engraftment (Gilbert et al., 2010). Besides FAPs’ role in modulating ECM remodeling and stiffness, others and we have shown that PDGFRα+ FAPs secrete various cytokines and growth factors, which directly induce myogenic cell proliferation and survival but may block their differentiation (Joe et al., 2010; Heredia et al., 2013; Biferali et al., 2019; Scott et al., 2019). These data strongly suggest that muscle-resident FAPs are critical to support MuSC and that their modulation is essential to provide pro-myogenic trophic functions.

However, chronic injury makes this heterogenic, multi-step, and coordinated response persistent instead of transient, priming the muscle milieu into an aberrant state known as fibro-fatty infiltration. In this context, exacerbated inflammation and accumulated scarring interferes with normal tissue function (Lieber and Ward, 2013; Dadgar et al., 2014; Ieronimakis et al., 2016; Buras et al., 2019). Indeed, we showed that after acute muscle injury, from day three onwards, macrophage-induced apoptosis clears the excess of PDGFRα+ FAPs (Figure 2, top) and that this process can be pharmacologically stimulated by nilotinib (Lemos et al., 2015; Fiore et al., 2016). Hence, modifying propensity of FAPs to engage in apoptosis might be a protective way to avoid excessive accumulation of these cells and fibrosis deposition in pathology. However, in pathological and chronic conditions, PDGFRα+ cells are over-activated and not efficiently cleared out, remaining in high numbers and differentiating toward multiple MSC lineages depending on the type and extension of the damage (Figure 2, bottom) (Lemos et al., 2015; Ieronimakis et al., 2016; Kopinke et al., 2017; Madaro et al., 2018; Malecova et al., 2018; Contreras et al., 2019a; Mázala et al., 2020).

Lastly, although most previous studies demonstrate that FAPs influence myogenic behavior and fate of MuSCs, the latter also exhibit trophic functions toward FAPs. Indeed, MuSCs and myotubes strongly reduce FAP adipogenic potential *in vitro* (Uezumi et al., 2010; Moratal et al., 2019). Co-culture experiments using primary myotubes suggest the presence of “unknown” factors in myogenic cells that inhibit the adipogenic differentiation of FAPs (Uezumi et al., 2010). Remarkably, the modulation of FAPs by myoblasts and myotubes is altered with aging and DMD as these myogenic cells lose their regulatory potential, which may explain the abundance of fat tissue in degenerated muscles (Moratal et al., 2019). Moratal et al. (2019) suggested that the myogenic progenitor secretome is crucial to regulate FAP lineages, and these “unknown” factors may influence PI3K-AKT, SMAD2, and GLI signaling pathways. These studies suggest that the myogenic lineage largely influences the plasticity of PDGFRα+ FAPs, albeit the cellular and molecular mechanisms remain elusive.

In sum, non-cell-autonomous mechanisms (e.g., muscle microenvironmental factors such as cell-to-cell contact, growth factors, cytokines/myokines, or matrix stiffness) also determine FAP dynamics. Hence, the destiny of FAP progeny, location, and abundance resolution within injured muscles can be modified extrinsically, aiming to boost skeletal muscle regeneration and enhanced tissue repair.

### Fibro-Adipogenic Progenitors and Immune Cells: A Sentinel Relationship

It is now well known that muscle-resident and non-resident immune cells, in particular macrophages (MPs), play essential roles in tissue homeostasis, regeneration, repair, and disease (reviewed in Tidball and Villalta, 2010; Vannella and Wynn, 2017; Perandini et al., 2018; Theret et al., 2019; Chazaud, 2020). Indeed, MPs are responsible for modulating MuSC proliferation and differentiation, with their depletion inducing extensive alteration of muscle regeneration following injury (Arnold et al., 2007; Lemos et al., 2015; Juban et al., 2018). Nevertheless, the cellular and molecular interactions of PDGFRα+ FAPs and macrophages at the resting state are unknown.

**FIGURE 3:**
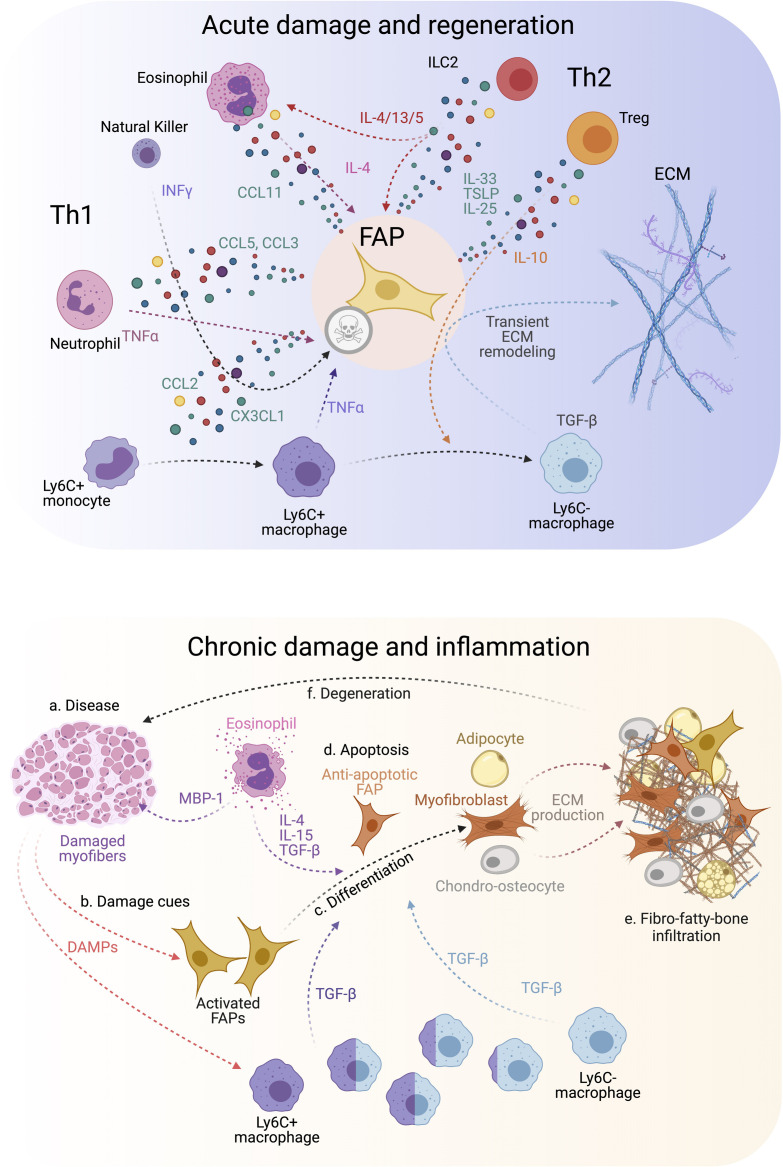
FAP-immune cell cross-talk and secretory flux in regeneration and disease. **(Top)** FAPs and several immune cell types are involved in regulating efficient muscle regeneration following injury. Th1 and Th2 immune cell populations characterize the immune response. This coordinated FAP-immune cell interaction occurs through the secretion of several pro-regenerative and inflammatory cues from both stromal compartments. All of this facilitates transient ECM remodeling by FAPs that promotes muscle regeneration. **(Bottom)** On the contrary, neuromuscular disorders and chronic trauma drive the muscle milieu into an aberrant state that usually supports repairing the damaged muscles. Gradually, persistent inflammation tip points the regenerative muscle potential toward muscle degeneration, causes over-activation of FAPs, increases TGF-β levels beyond a regenerative state, and leads to FAP-mediated tissue replacement by fibrous, fat and bone-like tissues. Owing to this, affected muscles reach a state of pathogenic positive feedback loop where damage-driven fibro-fatty-bone infiltration enhances tissue malfunctioning and failure.

Surprisingly, genetic lineage-depletion of PDGFRα+ cells does not affect muscle immune cell numbers at short (9–14 days) and long (9 months) periods post depletion in the absence of muscle injury (Wosczyna et al., 2019). However, their cellular cross-talk is better described after a single round of damage, chronic damage and in disease. After acute injury, blood circulating Ly6C+ monocytes infiltrate the damaged area in the first 24 h after injury and differentiate into Ly6C+ MPs. This crucial infiltration is chemokine-dependent (e.g., CCL2, CCL3, or CX3CL1) and due to the release of DAMPs (Damage-associated Molecular Pattern) cues (Figure 3, top) (Martinez et al., 2009; Brigitte et al., 2010; Sun et al., 2011). While many cells are involved in MP attraction, damage-activated FAPs express a complex cocktail of chemokines and cytokines 24 h after injury, thus establishing an early niche rich in inflammatory cues (Figure 3, top) (Mashinchian et al., 2018; Scott et al., 2019). This intricate primary response of FAPs during the initiation and formation of granulation tissue supports the notion that they are important components and modulators of the early immune response. Eventually, Ly6C+ MPs skew into a pro-restorative phenotype, associated with the downregulation of Ly6C (Figure 3, top) (Arnold et al., 2007; Varga et al., 2013). Besides, others and we described that Ly6C+ MPs have an early pro-apoptotic effect on PDGFRα+ FAPs through TNF-α production, keeping their number under control following injury resolution (Lemos et al., 2015; Juban et al., 2018). Therefore, posttraumatic inflammation stimulates local FAPs, leading to their activation, proliferation, accumulation, differentiation, and later resolution to steady-state conditions. These key concepts have been validated in the *Ccr2* knockout mice model (Lu et al., 2011a, b). The lack of infiltrating monocytes resulted in delayed PDGFRα+ FAP clearance and impaired muscle regeneration but increased fibro-fatty deposition (Lemos et al., 2015). These results demonstrate that infiltrating monocytes are required for proper FAP clearance and avoid excessive ECM deposition during damage resolution.

On the other hand, Ly6C- MPs secrete TGF-β, which activates pro-survival signaling pathways in FAPs and promotes their activation, ECM secretion, and myofibroblast differentiation (Figure 3, top) (Lemos et al., 2015; Juban et al., 2018; Contreras et al., 2019a, b, 2020; Stepien et al., 2020). Thus, the TNF-α/TGF-β balance needs to be carefully regulated since its disruption (lengthy exposure of TNF-α or increase of TGF-β levels) will induce drastic changes in FAP behavior, delaying skeletal muscle regeneration as often seen in degenerative myopathies (Figure 3, bottom) (Lemos et al., 2015; Muñoz-Cánoves and Serrano, 2015; Juban et al., 2018). The fact that intercellular communication within muscle is impaired during chronic damage might be one reason for tipping the system into dysregulated fibro-fatty remodeling and permanent scarring.

We postulate that chronic conditions and degenerative settings compromise the steady-state balance of FAPs. This latter hypothesis was recently corroborated by Saito et al. (2020). The authors, using an experimental autoimmune chronic inflammatory myopathy (CIM) model, showed that CIM-FAPs develop an anti-apoptotic phenotype compared to FAPs isolated from an acute injury model (intramuscular injection of BaCl2) (Figure 3, bottom) (Saito et al., 2020). Mechanistically, FAPs undergo senescence -characterized by the upregulation of *Ckn2a* and *Trp53* expression and the histone variant γH2A.X- upon acute muscle injury but not under CIM. Running exercise also induces rapid FAP senescence. Intriguingly, when subjecting the CIM mice to exercise combined with AICAR treatment, an AMP-activated kinase (AMPK) activator, both interventions restored muscle function, resulting in the induction of a pro-inflammatory and pro-apoptotic FAP phenotype that enhances muscle regeneration (Saito et al., 2020). AMPK has been previously shown to have a crucial role in macrophage polarization and MuSC self-renewal (Mounier et al., 2013; Theret et al., 2017; Juban et al., 2018). These results could explain the chronic accumulation of FAPs due to clearance deficiency as a consequence of senescence and apoptosis resistance in muscle pathologies, highlighting the potential of FAP-targeted therapeutic interventions employing exercise and AMPK activation for chronic muscle diseases.

Macrophages and FAPs lie near each other, especially after muscle trauma and pathology (Lemos et al., 2015; Contreras et al., 2016; Juban et al., 2018; Moratal et al., 2018). Aiming to decipher the interactions between MPs and FAPs, bone marrow-derived MPs can be treated *in vitro* with IL-4 to mimic an M2a/alternative activation (Murray et al., 2014), or IL1-β to phenocopy mild inflammatory MPs (M1) as found in muscles (Arnold et al., 2007). Interestingly, treatment of human FAPs with conditioned media obtained from IL-1β-polarized MPs reduced FAP adipogenesis via the TGF-β signaling pathway (Moratal et al., 2018). On the contrary, IL-4-polarized MPs enhanced FAP adipogenesis (Moratal et al., 2018). Recently, Stepien et al. (2020), using a combined fibrotic model of muscle damage, showed that decreasing the amount of infiltrating macrophage-derived TGF-β reduced fibrosis and proliferation of fibro-adipogenic progenitors and simultaneously enhanced muscle regeneration. To conclude, these studies performed in mouse and human muscles demonstrated that distinct subsets of macrophages could have opposite effects on FAP cellular states and fate, giving the possibility to explore regenerative outcomes by understanding their cellular cross-modulation.

MPs are not the only inflammatory cell type that interact with FAPs in regeneration and repair. Usually, neutrophils are the first cells that infiltrate the damaged tissue (Figure 2, top). Coming from either the blood or the fascia (Brigitte et al., 2010; Bentzinger et al., 2013), neutrophils also produce TNF-α, which may help initiate FAP clearance and resolve their increased numbers after damage (Pizza et al., 2005). Later, NK cells infiltrate the injured muscle, secrete INF-γ and stimulate myogenic cell proliferation (Cheng et al., 2008). Notably, the potential regulatory function of NK cells toward PDGFRα+ FAPs is unknown. In the same timeframe, eosinophils infiltrate the damaged muscle and secrete IL-4, which induces the proliferation of PDGFRα+ FAPs, providing in return pro-myogenic trophic effect on MuSCs (Figure 3, top) (Heredia et al., 2013). Remarkably, IL-4 inhibits the adipogenic differentiation of muscle FAPs *in vitro* and *in vivo* (Heredia et al., 2013; Dong et al., 2014). Like IL-4, IL-15 also promotes FAP proliferation and expansion while inhibiting their adipogenic commitment and fatty muscle deposition following acute damage. The enhanced accumulation of PDGFRα+ cells by intramuscular IL-15 triggers a transient increase of ECM, which seems to facilitate the regeneration of injured myofibers (Kang et al., 2018). Hence, the roles of ILs as therapeutic targets have opened new avenues in the search for effective muscle therapies to promote muscle regeneration and improve muscle atrophy associated with sarcopenia.

Type 2 immunity, due to the recent discovery of innate and type 2 lymphoid cells (ILC2), is a newly added component to our understanding of tissue homeostasis (Castiglioni et al., 2015; Tidball, 2017). ILC2 are tissue-resident cells and participate in the Th2 response by producing IL-4, IL-5, IL-13, and IL-9 (Licona-Limón et al., 2013). The release of IL-33, IL-25, and TSLP produced by MSCs after damage primarily recruits and activates ILC2 cells (Figure 3, top) (Yagi et al., 2014). Interestingly, muscle-resident MSCs (likely FAPs) from aged mice produced less IL-33 than their young counterparts, which delays ILC2 and Th2 inflammation (Kuswanto et al., 2016). Hence, FAP-derived IL-33 regulates the dynamics of muscle regulatory T-cells (Tregs) during muscle regeneration following acute injury (Figure 3, top). Owing to mammalian aging reduces the number of muscle-resident Tregs, it may explain the inefficiency of muscle regeneration and repair in the elderly population. Overall, ILC2s participates in tissue homeostasis, mostly in the lungs and the intestine (for ILC2 review, see Messing et al., 2020), but their function in muscle regeneration is underexplored. Muscle-resident Tregs (CD4+/FoxP3+ cells), however, limit the production of IFN-γ from NK cells, inhibiting local MP proliferation and pushing MP skewing during skeletal muscle regeneration (Figure 3, top) (Panduro et al., 2018). Indeed, Tregs-depleted muscle shows altered regeneration with increased inflammation and fibrosis, a typical phenotype of delayed Th1/Th2 skewing (Mounier et al., 2013; Panduro et al., 2018). Thus, disruption of the Th1/Th2 immunity impairs skeletal muscle homeostasis and regeneration.

Activated T cells express PD-1 (CD279) receptor, whereas MPs and dendritic cells present the ligands (PD-L1 and L2) at their cell surface (Zou, 2005). The PD-L1/PD-1 axis has been intensely investigated, mostly in cancer (Pardoll, 2012), whereas almost no information exists for muscle. T cell activation requires the PD-1/PD-L1 interaction, and its inhibition induces T cell over-activation, leading to a Th2 inflammatory response (Pardoll, 2012). While those inhibitors have created significant opportunities for cancer therapies, their use often causes under-reported cardiac and skeletal myopathies, leading to lethal conditions in some cases (Johnson et al., 2016; Zimmer et al., 2016; Matas-García et al., 2020). Although T cells participate in skeletal muscle regeneration (Deyhle and Hyldahl, 2018), the mechanisms of how T cell over-activation can lead to muscle defects and myopathies are far from understood. Moreover, how NK cells, T cells, and ILC2s directly affect the fate and activities of muscle PDGFRα+ FAPs is unclear. Previous studies have mostly focused on MuSC behavior in various immune-deficient mouse models without concentrating on the interstitial compartment. Accordingly, future work should address these questions and keep the muscle research field busy for the next few years.

## Fibro-Adipogenic Progenitors as Perivascular Cells

Skeletal muscle is highly vascularized and adapted for enormous blood flow rate changes in response to exercise (Korthuis, 2011). Given the high amount of muscle mass that the human body encompasses, skeletal muscles’ metabolic and endocrine functions are tightly linked to its vascular density (Olfert et al., 2016). Endothelial cells, pericytes, and smooth muscle cells form the vascular muscle milieu – capillaries, arterioles, arteries, and venules. Briefly, different populations of cells organized in concentric layers constitute the cellular wall of blood vessels: endothelial cells (known as tunica intima), mural cells of the tunica media (pericytes in capillaries or vascular smooth muscle in larger vessels), and adventitial or perivascular fibroblasts (tunica adventitia) (Cattaneo et al., 2020). As the role of skeletal muscle vasculature in homeostasis and disease has been reviewed elsewhere (Cooke and Losordo, 2015; Latroche et al., 2015a), here we focus on the participation of muscle-resident FAPs in modulating vascular remodeling.

Many PDGFRα+ FAPs localize circumferentially around vessels in muscles but reside outside the basal membrane of vascular cells (Joe et al., 2010; Uezumi et al., 2010, 2021; Scott et al., 2019). Thus, PDGFRα+ FAPs are a significant proportion of perivascular stromal cells residing in the vascular wall’s tunica adventitia. Indeed, when muscle-resident mesenchymal cells are lineage traced based on the expression of *Hypermethylated in cancer 1* (*Hic1*, also known as HIC ZBTB Transcriptional Repressor 1), the vast majority of these cells are FAPs (Scott et al., 2019). HIC1 is an epigenetically-regulated zinc-finger transcription factor that acts as a transcriptional repressor, regulating cell growth, proliferation, and its dysregulated expression associates with several malignant disorders and cancer (Chen et al., 2003; Zheng et al., 2012; Scott et al., 2019; Contreras, 2020; Soliman et al., 2020). Only a small proportion (5%) of muscle-resident HIC1+ cells that are SCA-1 negative, identified as tenogenic cells and pericytes (Scott et al., 2019). Whether HIC1+ progenitors could give rise to vascular RGS5+ pericytes is unknown, although the relative contribution of HIC1+ cells to vascular pericytes suggests a common hierarchy progenitor in adult muscles that may divide the perivascular cell ontogeny early during development (Contreras, 2020). However, the lineage relationship and ontology of HIC1+ cells with tenogenic cells and pericytes remains unclear.

In humans, resistance training is associated with increased proliferative PDGFRα+ FAPs but a decreased abundance of pericytes (Farup et al., 2015). Hence, the active remodeling of the stromal compartment following exercise results in physiologically relevant cues for satellite cell expansion post-training, suggesting that physical activity may actively promote a healthy muscle niche to restrain age-related development IMAT and fibrosis during sarcopenia (Collao et al., 2020). Addressing these significant questions related to the possible roles of FAPs in skeletal muscle during exercise and sarcopenia will keep the field busy for the next few years.

While myogenesis and angiogenesis seem to be primarily affected by the MP inflammatory and secretory status during muscle regeneration (Latroche et al., 2017), the role of PDGFRα+ FAPs in neo-angiogenesis are understudied. Indeed, co-culture of human umbilical vein endothelial cells (HUVECs) with human dermal fibroblasts does not affect capillaries’ formation *in vitro* or *in vivo* angiogenesis (Latroche et al., 2017). However, dermal fibroblasts are significantly different from muscle FAPs. Santini et al. (2020) recently confirmed that PDGFRα+ cells are found in a pericytic position in muscles, corroborating previous findings of FAPs being perivascular cells (Joe et al., 2010; Uezumi et al., 2010, 2021; Scott et al., 2019). The authors also showed that genetic ablation of PDGFRα+ FAPs disrupts vessel organization and impairs revascularization, which leads to increased fibrosis and muscle damage after hind limb ischemia (HLI) (Santini et al., 2020). No ablation-dependent changes were seen in the absence of damage. Thus, this study established that muscle-resident FAPs are necessary for effective revascularization upon ischemia, although the regenerative cues or factor(s) they synthesized remain underexplored. An intriguing possibility is the participation of vascular growth factors, like angiopoietins or vascular endothelial growth factor (VEGF), known for regulating embryonic and postnatal angiogenesis. These secreted proteins are signaling hubs that promote blood vessels’ growth and, within muscles, seem to be expressed by endothelial cells, MuSCs, and FAPs (Verma et al., 2018). The reason for the redundancy of expression is unknown, but we hypothesize that they could participate in autocrine signaling loops in a cell-type-specific fine-tuned manner. Utilizing a mouse model of heterotopic ossification (HO), Hwang et al. (2019) showed that mesenchymal progenitors, rather than endothelial cells, dynamically regulate the expression of *Vegfa* following trauma. The authors also showed that *Prx*-lineage derived VEGFA is necessary to induce ectopic bone formation (Hwang et al., 2019). These novel findings position muscle-resident mesenchymal progenitors (i.e., FAPs) as a critical source of vascular and angiogenesis factors in severe trauma and disease.

Although separated by their respective basal laminas, MuSCs and endothelial cells are in close contact (Christov et al., 2007; Verma et al., 2018), and neo-angiogenesis in response to muscle trauma is essential to supply blood to the newly formed myofibers efficiently. While increased vascular density leads to augmented MuSC numbers and ameliorates DMD myopathology (Verma et al., 2010), mostly by increasing VEGF and FGF (Fibroblast Growth Factor) availability (Neuhaus et al., 2003; Deasy et al., 2009); altered muscle vasculature negatively impacts tissue maintenance, vascular tone, myofiber type density, and myogenic progenitor homeostasis (Matsakas et al., 2013). Indeed, blood vessel density and vascular integrity around myofibers are reduced in a number of mice models of neuromuscular disorders (Latroche et al., 2015b; Valle-Tenney et al., 2020). Hence, decreased blood perfusion of the skeletal muscle could contribute to its regeneration and revascularization defects. Interestingly, in dermatomyositis, loss of capillaries is associated with a decrease in the number of satellite cells in the affected area, in the absence of local muscle damage (Gitiaux et al., 2013). This interdependent relationship emphasizes the importance of the interaction between the two cell types. However, the exact interplay between MuSCs and endothelial cells *in vivo* needs further examination. Furthermore, the cellular interactions and the extension of the association between the vasculature and FAPs are practically unknown.

Muscle injury reduces the vascular network (Hardy et al., 2016; Morton et al., 2019) and leads to increased hypoxia and hypoxia-inducible factor 1a (HIF-1α), suggesting that injured muscles are in an hypoxic state (Drouin et al., 2019; Valle-Tenney et al., 2020). Recently, Drouin et al. (2019) showed that muscle damage-induced hypoxia promotes the activation and proliferation of SCA-1+ FAPs. Hypoxia reduces the adipogenic differentiation of FAPs, but it promotes their osteogenic differentiation. Accordingly, *in vitro* simulated hypoxia alters the fate of muscle-resident FAPs. Sustained activation of SMAD1/5/8 and induced expression of BMP9 appear to contribute to these hypoxia-mediated effects on FAP behavior (Drouin et al., 2019). Thus, loss of blood perfusion and reduced oxygen supply affect FAP fate. Remarkably, genetic loss of *Hif1a* in mesenchymal progenitors (*Prx* expressing lineage) prevents HO formation (Agarwal et al., 2016), which suggests that HIF-1α could participate in regulating the chondrogenic and osteogenic fate of mesenchymal progenitor cells. Nevertheless, the specific role(s) of HIFs (e.g., HIF-1α) in modulating the activities of PDGFRα+ cells remain unrevealed to date.

## Fibro-Adipogenic Progenitors as Pathological Drivers of Muscle Fibrosis and Intramuscular Fat Accumulation in Pathology and Disease

Fibrosis is the most common degenerative outcome of several diseases characterized by chronic damage and inflammatory response, can affect any organ, and accounts for about half of all deaths in the industrialized world (Henderson et al., 2020). Progressive and excessive ECM accumulation in connective tissue, also called CT hyperplasia, characterizes fibrosis (reviewed in Serrano et al., 2011; Lieber and Ward, 2013; Henderson et al., 2020). The principal components of muscle CT are the ECM and its stromal cells that continuously reshape this complex structural and supportive microenvironment. Muscle pathological fibrosis is different from the transient physiological “fibrosis” or enhanced ECM deposition that follows a single round of injury (Smith and Barton, 2018). This physiological fibrosis is required to rebuild lost muscle architecture and facilitates muscle regeneration. However, under chronic damage, inflammatory-activated fibroblasts and myofibroblasts express large amounts of ECM proteins resulting in the replacement of myofibers with wound scar, thereby leading to muscle failure and even death (Fadic et al., 2006; Pakshir and Hinz, 2018). Hence, although active ECM remodeling is essential for tissue regeneration and repair, its exacerbated and disorganized accumulation during pathology often leads to tissue malfunctioning (Borthwick et al., 2013). Among the most affected tissues by fibrosis are the skin, liver, lungs, heart, kidney, eye, pancreas, intestine, brain, bone marrow, and skeletal muscles (Wynn and Ramalingam, 2012). Given the importance of scar formation in several tissues in pathology, disease, and aging (Brack and Rando, 2008; Grounds, 2008; Kharraz et al., 2014; Purslow, 2020), it is perhaps surprising that the biology of PDGFRα+ FAPs is not well understood yet.

The *in vivo* fibrogenic potential of PDGFRα+ cells was initially confirmed by PDGFRα^*H2BEGFP*^+ cell transplantation into irradiated skeletal muscle after toxin-induced muscle injury (Hamilton et al., 2003; Uezumi et al., 2011). Transplanted PDGFRα^*H2BEGFP*+^ cells did not form myofibers but accumulated in ECM-rich areas within the muscle interstitium. PDGFRα+ progenitors also expand and gather in muscle fibrous-rich areas of Duchenne muscular dystrophy (DMD) patients and dystrophic mdx mice models (Uezumi et al., 2014; Ieronimakis et al., 2013; Lemos et al., 2015; El Agha et al., 2017; Kopinke et al., 2017; Contreras et al., 2019a; Mázala et al., 2020). Fibrogenic FAPs can also be identified by expression of *Collagen I* (Theret et al., 2021), although other muscle-resident cells like SCA-1 negative and MuSCs also express basal levels of this fibrillar Collagen gene (Chapman et al., 2016). Others and we have proposed that a significant proportion of activated fibroblasts and myofibroblasts are generated by PDGFRα+ FAPs upon injury or *in vitro* through the stimulation of FAPs with profibrotic cytokines (e.g., TGF-β) combined with serial passaging in normoxic atmosphere (Contreras et al., 2019a, b, 2020; Santini et al., 2020; Stepien et al., 2020). Interestingly, MYOSTATIN, a secreted TGF-β family member that negatively regulates myofiber size, also induces FAP proliferation, myofibroblast differentiation, and muscle fibrosis (Zhao et al., 2008; Dong et al., 2017). Hence, these pro-fibrotic cues regulate how and when PDGFRα^+^ progenitors expand and gather in muscle fibrous-rich areas.

**FIGURE 4:**
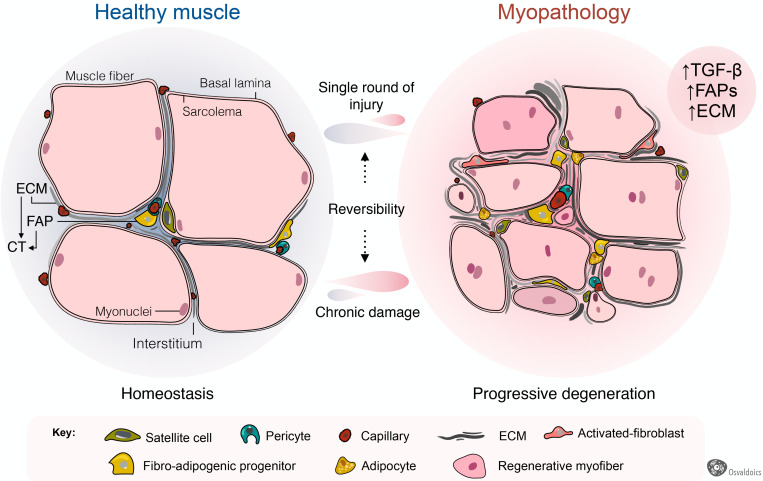
Fibro-adipogenic progenitors as central drivers of muscle pathology and fibro-fatty infiltration. Skeletal muscles have high regenerative capabilities after a single round of injury, which progressively diminishes following chronic damage. Hence, chronic injury primes the tissue into a state of progressive degeneration. In homeostasis, a high proportion of fibro-adipogenic progenitors are perivascular cells and remain quiescent. Injury activates FAPs and induces their expansion and differentiation into several mesenchymal lineages, including activated fibroblasts, adipocytes, and bone-like cells. Activated FAPs and their lineage derived fibroblasts produce increased amounts of extracellular matrix (ECM), leading to connective tissue hyperplasia and poorer outcomes for patients. In several traumas and pathology, the adipogenic differentiation of FAPs also impairs muscle function and integrity. Muscle degeneration associates with an increased number of FAPs, activated TGF-β signaling and enhanced fibrosis. Chondrogenic and osteogenic FAP differentiation are not depicted to simplify the cartoon.

Fibrosis is a common hallmark of several congenital muscular dystrophies, and notably, FAPs are pathologically dysregulated in most of them (Figure 4). These include mouse models of *Collagen VI*- (Noguchi et al., 2017; Mohassel et al., 2018), human DMD (Uezumi et al., 2014) and mouse models of DMD (Box 1) (Uezumi et al., 2011; Mozzetta et al., 2013; Lemos et al., 2015; Contreras et al., 2016, 2020; Ieronimakis et al., 2016; Kopinke et al., 2017; Mázala et al., 2020; Reggio et al., 2020; Giuliani et al., 2021), Facioscapulohumeral dystrophy (FSHD) (Bosnakovski et al., 2017, 2020), Limb-girdle muscular dystrophy (LGMD) (Hogarth et al., 2019), and ALS neuromuscular disease (Gonzalez et al., 2017; Madaro et al., 2018). Increased muscle fibrosis and FAP expansion also occur after single and repeated cycles of intramuscular BaCl_2_, notexin, cardiotoxin, or glycerol administrations (Uezumi et al., 2010; Dadgar et al., 2014; Pessina et al., 2014; Contreras et al., 2016, 2020; Theret et al., 2021), vastus medialis muscles of compromised knee osteoarthritis patients (Ikemoto-Uezumi et al., 2017), human anterior cruciate ligament injuries (Fry et al., 2017), induced hindlimb ischemia (Santini et al., 2020), degenerated muscles of type 2 diabetic patients (Farup et al., 2020), obesity-mediated diaphragm dysfunction of chronic high-fat diet-fed mice (Buras et al., 2019), and surgical muscle traumas including denervation and laceration (Brandan et al., 1992; Pessina et al., 2014; Contreras et al., 2016; Madaro et al., 2018; Rebolledo et al., 2019). Muscle-resident FAPs also increase and accumulate in a muscle fibrosis model of chronic kidney disease (Dong et al., 2017) and chronic kidney disease human patients (Abramowitz et al., 2018), and are accompanied by enhanced muscle atrophy and FAP-mediated adipogenesis (Hu et al., 2019). In each of these pathologies and models of muscle injury and scarring, the extension and degree of fibrosis largely depend on the type and extension of damage and myodegeneration. Consequently, we reason that muscle scarring originates from PDGFRα+ FAPs (Figure 4).

**Box 1. Participation of muscle fibro-adipogenic progenitors in DMD pathology.**

DMD is a severe human myopathy without cure to date. It is caused by mutations in the *DMD* gene encoding DYSTROPHIN and characterized by muscular degeneration in which progressive loss of muscle mass and weakness are expected consequences (Hoffman et al., 1987; Wallace and McNally, 2009). Myonecrosis, sarcolemmal disorganization, inflammation, and the accumulation of fibro-fatty scar tissue are observed early in DMD and increase with age (Cáceres et al., 2000; Porter et al., 2002; Reed and Bloch, 2005; Serrano et al., 2011; Grounds et al., 2020). Remarkably, FAPs generate activated fibroblasts, myofibroblasts, adipocytes, and bone-like cell progeny in muscular dystrophy, albeit to different extents depending on the degree of damage, inflammation and degeneration (Uezumi et al., 2011, 2014; Lemos et al., 2015; Contreras et al., 2016, 2019a; Ieronimakis et al., 2016; Kopinke et al., 2017; Eisner et al., 2020; Mázala et al., 2020). As known for other muscle-resident cells, the exit of FAPs from quiescence and their entry into proliferative and differentiation programs are not only finely tuned by microenvironmental cues (Uezumi et al., 2010; Heredia et al., 2013; Moratal et al., 2018, 2019) but also through intrinsic molecular mechanisms like epigenetic HDAC-dependent pathways (Mozzetta et al., 2013; Saccone et al., 2014; Sandonà et al., 2020) or Hic1-dependent quiescence maintenance (Scott et al., 2019; Contreras, 2020).

Adult skeletal muscles fibrosis is also associated with adipose tissue accumulation following a single-round of injury (Pisani et al., 2010; Biltz and Meyer, 2017; Biltz et al., 2020; Xu et al., 2021) and in several myopathies, including DMD (Box 1) (Uezumi et al., 2011, 2014; Kopinke et al., 2017), LGMD (Hogarth et al., 2019), and after rotator cuff tears (Davies et al., 2016; Liu et al., 2016; Kang et al., 2018; Lee et al., 2020a). Remarkably, increased numbers of PDGFRα+ FAPs are found in many of these myopathologies, being responsible, at least at some extent, for exacerbated fat deposition (Figure 4). Thus, growing evidence suggests that FAPs are, to some extent, drivers of the muscle loss and replacement by a non-contractile scar in disease and pathology (Box 1). Intriguingly, FAPs are the cells in charge of both physiological and pathological ECM and fat accumulation. However, the extrinsic or intrinsic mechanisms regulating FAP activation, plasticity, and fate during muscle loss progression and fibro-fatty replacement are still underexplored.

Remarkably, the passaging of PDGFRα+ FAPs in plastic dishes and 20% oxygen is sufficient to induce their activation, acquisition of myofibroblast features, and loss of PDGFRα expression (Contreras et al., 2019b; Santini et al., 2020). Recently, Santini et al. (2020) injected PDGFRα+-derived activated fibroblasts and myofibroblasts into nude mice after hindlimb ischemia to study whether differentiated myofibroblasts contribute to fibrosis and impair vessel maturation. Following HLI surgery, myofibroblast’s transplantation differentiated from PDGFRα+ FAPs increased vessel leakage, impaired regeneration, and increased fibrosis compared to PBS-injected nude mice. The injected cells survived for at least 3 weeks within the HLI damaged tissue and remained associated with ECM-rich foci near myofibers (Santini et al., 2020). Finally, by using a clonal Brainbow lineage tracing strategy, Santini et al. (2020) also demonstrated that most of the progeny of PDGFRα+ FAPs is associated with vessels and fibrotic tissues. Concurrently, a smaller proportion was identified in adipogenic regions following HLI, which indicates that ischemia primarily primes their fate toward a fibrogenic phenotype. In summary, PDGFRα+ FAPs are required for muscle repair after HLI by actively remodeling ECM and supporting neovascularization.

Potentially, PDGFRα+ lineage-derived myofibroblasts are not the only players in stromal fibroproliferative-disorders and scarring. The participation of other tissue-resident perivascular cells (e.g., pericytes) or immune cells (e.g., MPs) to matrix remodeling upon injury, and the establishment of permanent scars, cannot be excluded (Henderson et al., 2013; Mederacke et al., 2013; Lemos and Duffield, 2018; Van Caam et al., 2018; Pakshir et al., 2020). For example, in tissues different from skeletal muscle (e.g., kidney), perivascular ADAM12+ progenitors that differentiate toward a myofibroblast-like phenotype when treated with TGF-β also contribute to tissue regeneration and fibrotic scarring following damage (Armulik et al., 2011; Lebleu et al., 2013; Birbrair et al., 2014). In skeletal muscles, Dulauroy et al. (2012) added a piece to the puzzle by characterizing both fetal and adult ADAM12+ progenitors. Postnatally, ADAM12+ cells represent two distinct populations of cells; NCC-derived Schwann cells (S100+) and perivascular PDGFRβ expressing pericytes (Dulauroy et al., 2012). Interestingly, even when tissue injury reactivates both subsets of ADAM12+ cells, and they expand accordingly within the muscle interstitium, only ADAM12^+^-PDGFRβ^+^ perivascular cells -likely pericytes or a FAP subpopulation- but not NCC-derived cells generate myofibroblasts (Dulauroy et al., 2012). Thus, upon acute muscle injury, the ADAM12+-derived myofibroblasts are of the mesenchymal lineage but not of the Schwann cell lineage or NCCs-derived. Since ADAM12+ progenitors do not differentiate toward other MSC derivatives such as adipocytes, they may have lineage restrictions or represent a subset of pre-committed FAPs into a fibrogenic fate (Dulauroy et al., 2012). Likely, PDGFRα+ and ADAM12+ cells merge their lineages to some extent, but PDGFRα+ FAPs are a more broad-spectrum multipotent population.

Similarly, perivascular cells expressing the zinc finger protein Gli1 (also known as glioma-associated oncogene 1) undergo proliferative expansion and generate myofibroblasts after injuring kidney, lung, liver, and heart (Kramann et al., 2015). All the Gli1+ cells with CFU-F properties express PDGFRβ, but only a tiny fraction of PDGFRβ+ cells in tissues and organs are Gli1 expressing cells (Kramann et al., 2015). There is also the possibility that muscle-resident Gli1+ cells are a subpopulation of muscle FAP cells. This idea was recently demonstrated by Yao et al. (2021). Lineage tracing of muscle-resident Gli1 expressing cells demonstrated that Gli1+ cells correspond to a small subpopulation -about 10 to 15%- of the total Sca1+/PDGFRα+ cells in homeostasis (Yao et al., 2021). However, Gli1+ cells rapidly expand following notexin muscle injury, and their relative proportion increases to about 40% of the total FAPs 3 days post damage. Accordingly, Gli1+ FAPs possess higher clonogenicity (CFU-F frequency) and reduced adipogenesis than Gli1 negative FAPs. Remarkably, the authors also showed that the genetic ablation of Gli1+ FAPs delays muscle regeneration. Finally, utilizing single-cell profiling, the authors suggested that Gli1+ FAPs preferentially express pro-myogenic and anti-adipogenic genes than Gli1-FAPs. These results strongly suggest differential proliferative and fate capabilities within distinct muscle-resident FAP subpopulations.

## Cellular Origins of Muscle Heterotopic Ossification and Calcification in Myopathologies

In normal conditions, muscles do not accumulate ectopic bone or intramuscular calcium deposits. However, muscles of DMD patients and the *mdx* mouse model exhibit intramuscular calcium deposits and bone (Bozycki et al., 2018). When stimulated, PDGFRα+ FAPs efficiently differentiate into the osteogenic lineage *in vitro* (Uezumi et al., 2010; Oishi et al., 2013). An increasing body of evidence suggests that murine and human stromal cells with osteogenic properties accumulate in muscles following injury, BMP2 intramuscular injections, and transplantation (Leblanc et al., 2011; Wosczyna et al., 2012; Oishi et al., 2013; Eisner et al., 2020). These muscle-resident cells also contribute to bone fracture repair in mice (Glass et al., 2011). It has been previously suggested that these progenitors are the primary source of abnormal formation of ectopic bone in muscles (Dey et al., 2016; Lees-Shepard et al., 2018b). Remarkably, the cells responsible for the rapid expansion of ectopic bone in fibrodysplasia ossificans progressiva (FOP), a genetic condition caused by mutations in ACTVR1 that alter the intracellular signaling pathways triggered by ACTIVIN-A, have been identified as FAPs (Dey et al., 2016; Upadhyay et al., 2017; Lees-Shepard et al., 2018a, b). By utilizing lineage-tracing experiments and a BMP2-induced HO, we recently showed that muscle-resident PDGFRα+ FAPs is the predominant cellular source of muscle ectopic ossification (Eisner et al., 2020). Using a parabiosis mice model, we also showed that muscle-resident PDGFRα+ FAPs, and not bone-marrow-derived PDGFRα+ cells, are the cellular source of heterotopic ossicles. Remarkably, DMD primes a osteogenic gene signature in these progenitor cells (Eisner et al., 2020). In addition, Mázala et al. (2020) showed that FAPs accumulate and deposit calcium-rich structures (bone) in higher proportions in a severe muscle disease DMD model D2-*mdx* mice (DBA/2J genetic background) than the mild dystrophic *mdx* mice (C57BL/10 genetic background). The authors also reported that the osteogenic differentiation of FAPs correlates with the level of muscle damage and TGF-β signaling (Mázala et al., 2020). Remarkably, intramuscular injections of a TGF-β signaling pathway inhibitor (ITD-1) blocked FAP accumulation and reduced fibro-calcification and muscle degeneration (Mázala et al., 2020). Others and we supported these findings, showing that FAPs expand and accumulate in relation to the type of muscle injury, TGF-β signaling, and wound scar (Uezumi et al., 2010, 2014; Lemos et al., 2015; Contreras et al., 2016, 2019a; Stepien et al., 2020). However, the reparative cues and signals that regulate bone differentiation of FAPs are unknown.

Collectively, these data strongly suggest three important conclusions: (1) The loss of regenerative muscle potential during myodegeneration leads to a chronic expansion of resident PDGFRα+ FAPs, elevated pro-fibrotic signals, and increased scarring; (2) PDGFRα+ precursor cells display MSC-like multipotency within the osteocyte, chondrocyte, activated fibroblast and myofibroblast, and adipocyte lineages; (3) The muscle microenvironment primarily dictates FAP developmental fate throughout damage-associated cues. In summary, FAP activity and responses are highly contextual, which suggests that signals emanating from the local niche determine their multi-lineage-fate.

Since most of the work related to FAPs biology used models of single or repeated rounds of injury, we propose that further studies should investigate the role of FAPs in atrophy-related pathologies such as aging-related sarcopenia, myasthenia gravis, polytrauma, and neuromuscular disorders.

## Emerging Roles of Fibro-Adipogenic Progenitors in Neuromuscular Disorders and Nerve Trauma

### Altered FAP Activation, Fate, and Behavior in Neuromuscular Disorders

The behavior and plasticity of muscle-resident PDGFRα+ FAPs in non-inflammatory or mild-inflammatory muscle perturbations such as mechanical denervation, neuropathies, amyotrophic lateral sclerosis (ALS), spinal cord injury, and spinal muscular atrophy (SMA) have not been explored until very recently. A typical result of these degenerative neuromuscular disorders is muscle denervation, resulting in the loss of electrical nerve transmission and nerve-associated cues and supplies. Failure of muscle innervation can result from physical compression of nerves, toxins, trauma, diseases, aging, or surgical interventions (Slater and Schiaffino, 2008). The significant outcomes of sustained muscle denervation are the partial or complete loss of movement and control of the denervated muscle group, and lastly, increased muscle atrophy and loss of tissue homeostasis (Batt et al., 2006). Along with capillary density reduction, skeletal muscle also undergoes an inexorable course of myofiber replacement by fibro-fatty tissue in the absence of innervation (Gatchalian et al., 1989; Fadic et al., 1990; Dulor et al., 1998; Borisov et al., 2001; Carlson, 2014). Remarkably, complex tissue regeneration following digit tip amputation requires innervation (Johnston et al., 2016; Carr et al., 2019).

Denervation also hinders proper skeletal muscle regeneration upon toxin-induced injury, and transection of the sciatic nerve worsens the repair of dystrophic hind limb muscles in mice (Pessina et al., 2014). Interestingly, compared to models of acute or chronic muscle injuries, denervation does not activate a noticeable initial inflammatory response or abundant mononuclear infiltration of immune cells (Contreras et al., 2016; Madaro et al., 2018; Rebolledo et al., 2019). PDGFRα+ FAPs proliferate and expand under these mild models of skeletal muscle damage, although to a lesser extent than in acute injuries (Contreras et al., 2016, 2019a; Malecova et al., 2018; Rebolledo et al., 2019). Sciatic nerve transection causes an early sustained activation and expansion of hind limb PDGFRα+ FAPs, correlating with increased TGF-β levels and ECM production and deposition from day two after peripheral nerve injury (Gatchalian et al., 1989; Contreras et al., 2016, 2019a; Rebolledo et al., 2019).

Additionally, we showed that FAPs accumulate in ECM-rich areas of atrophied muscles of the post-symptomatic ALS mice model (hSOD1^*G93A*^) (Gonzalez et al., 2017). Therefore, we can arguably speculate that PDGFRα+ cells are responsive to weak damage stimuli and elicit early responses to maintain tissue homeostasis and regeneration upon nerve-related perturbations. Central to this idea, Madaro et al. (2018) demonstrated that PDGFRα+ cells from denervated muscles have a different gene signature than cardiotoxin-derived cells, suggesting that FAP cellular and molecular responses differ from one model of injury to another. They also showed that *in vivo* treatment with a neutralizing IL-6 antibody or the inhibition of STAT3-dependent signaling using a pharmacological inhibitor prevented tissue atrophy and fibrosis in different mice models of muscle denervation. Thus, their work suggests that a FAP-driven cascade that involves IL-6-STAT3 activation can act as an initiator of muscle dysfunction by promoting fibrosis, and possibly myofiber atrophy, in denervated muscles, spinal cord injury, SMA, and ALS. These discoveries support the notion that PDGFRα+ FAPs act as tissue-disturbance sensors and open new avenues for developing cellular specific targets for non-inflammatory or mild-inflammatory neuromuscular disorders and for treating denervation-induced fibro-fatty deposition.

Although Madaro et al. (2018) observed no changes in the number of muscle infiltrating CD45+ cells after denervation, perhaps it would be interesting to evaluate the functional state of inflammatory cells such as neutrophils, eosinophils, natural killer cells, etc., which we known are required for proper skeletal muscle regeneration. Indeed, we think that regardless of whether there is or not an alteration in the number of these immune cells, changes in their activation status could be sufficient to induce an effect on the behavior and plasticity of FAPs. Moreover, we hypothesize that even a small level of immune activation in muscles after denervation (Hanwei and Zhao, 2010) or ALS (Gasco et al., 2017; Wang et al., 2017) could affect the quiescence of PDGFRα+ FAPs. Future work should concentrate on studying the role of the immune compartment or inflammatory-related pathways on neuromuscular disorders’ pathological features and their influence on FAP activation and fate.

We also raised the old-fashion hypothesis that a response of FAPs to denervation could be necessary to support efficient reinnervation. FAPs may assist and coordinate the attraction of axons toward specialized interstitial sites near to denervated myofibers -perisynaptic regions of neuromuscular junctions (NMJ)- by producing deposits of adhesive ECM molecules (e.g., n-cam, tenascin, fibronectin, and proteoglycans) (Covault and Sanes, 1985; Gatchalian et al., 1989; Court et al., 2008). The regenerative role of these nerve-associated cells is important since they are active contributors to the amputated digit tip (Johnston et al., 2016; Carr et al., 2019) and actively regulate muscle-nerve homeostasis, NMJ integrity, and Schwann cell behavior (Uezumi et al., 2021). Thus, upcoming research should also identify the cues involved in FAP activation after nerve trauma or neuromuscular disorders. We speculate that alterations of NMJ architecture, loss of nerve-associated factors, or even the absence of electrical stimulation could be playing a central role in determining the early activation of the stromal compartment in neuromuscular disorders.

### FAP-Mediated Fibro-Fatty Degeneration in Rotator Cuff Tears

The rotator cuff is an array of different muscles and tendons surrounding the shoulder joint giving support to the upper arm and shoulder. Rotator cuff tear (RCT) injury is a common cause of pain and disability among adults and the elderly, leading to increased shoulder dysfunction prevalence (Yamamoto et al., 2010). Rotator cuff damage can result from either acute or chronic injury to the shoulder or progressive degeneration of the tendon and myotendinous tissue, the magnitude of which influences the extension and severity of rotator cuff disease (Laron et al., 2012). Functional impairment of the cuff muscles following RCTs is associated with the degree of muscular degeneration, atrophy, and fibro-fatty infiltration (Meyer et al., 2004). These myodegeneration hallmarks increase accordingly with the size of tears and aging (Laron et al., 2012). The functional and clinical outcomes of patients undergoing RCT surgical repair also correlates with fibrosis, fatty infiltration, and atrophy of the *spinatus* muscles (Barry et al., 2013). Thus, poor functional outcomes result from massive tears in RCT patients who develop fibro-fatty deposition and muscle atrophy. No cure or effective treatments for these muscle pathologies are available to date.

Recent studies describe an increase of FAP number within RCT affected muscles. Indeed, FAPs and TIE2+ cells were initially proposed to be the cellular source of fibrosis and fat during this sustained muscle disease (Liu et al., 2016). Recently, the work led by Jensen et al. (2018) confirmed that the subpopulation of PDGFRβ+ cells that were PDGFRα+ contribute to fibro-fatty deposition in a severe murine model of RCT. At the molecular level, increased TGF-β signaling and ECM expression are associated with muscle pathology in RCT murine models (Liu et al., 2014). As previously described, damage-induced TGF-β promotes fibrosis by modulating the behavior, fate, and plasticity of muscle-resident PDGFRα+ FAPs. In this context, Davies et al. (2016) demonstrated that TGF-β signaling inhibition utilizing the SB431542 drug reduced fibro-fatty infiltration of RCT muscles. A significant reduction of PDGFRα+ FAPs preceded the decreased fibro-fatty accumulation after *in vivo* treatment with SB431542, which again points to the pro-survival and mitogenic actions of the TGF-β pathway in FAPs (Davies et al., 2016).

Similarly, Shirasawa et al. (2017), by generating a new RCT mouse model of enhanced fibro-fatty infiltration, observed that denervation dramatically exacerbated RCT muscle pathology of the transected suprascapular muscle. The authors confirmed that FAPs expand in this novel RCT model and reported that the treatment with imatinib, a first-generation tyrosine kinase inhibitor, significantly diminishes muscle fatty infiltration (Shirasawa et al., 2017). These results agree with our previous studies showing that nilotinib ameliorates muscular dystrophy disease by inducing FAP death (Lemos et al., 2015; Fiore et al., 2016). TGF-β and PDGFRα pathways are two central transduction cascades governing connective tissue biology and fibrosis (Kim et al., 2018; Smith and Barton, 2018; Contreras et al., 2019a). Interestingly, both imatinib and nilotinib are potent inhibitors of PDGFRα and TGF-β signaling and the downstream mediator mitogen-activated protein kinase (MAPK) p38 (Lemos et al., 2015; Contreras et al., 2018; Theret et al., 2021). Besides, we recently reported a functional cross-talk between TGF-β and PDGFRα involved in regulating the biology of tissue-resident PDGFRα+ cells (Contreras et al., 2019a). TGF-β negatively regulated the expression of PDGFRα in these cells, whereas PDGFRα signaling modulated TGF-β-mediated cellular and molecular effects (Contreras et al., 2019a). As for TGF-β, ILs are also known for being inflammatory mediators and appear to participate in RCT pathology. The expression of IL-15 positively correlates with the severity of ECM deposition, fibrosis, and FAP expansion in patients with chronic RCT (Kang et al., 2018). In conjunction, these data suggest active intercellular communication between immune cells and PDGFRα+ lineage cells in RCT disease. However, how interactions between these two populations of cells regulate RCT disease and fibro-fatty development remains unknown and warrants further investigations.

Interestingly, the distribution of PDGFRα+ FAPs is non-homogeneous between different muscle groups, and their anatomical locations seem to determine FAP expansion and behavior upon injury (Contreras et al., 2019a; Lee et al., 2020a). Interestingly, the RC *spinatus* muscle contains more FAPs per gram of tissue, and these cells show enhanced adipogenic and proliferative capacity compared to tibialis anterior- or gastrocnemius-derived FAPs, possibly explaining the increased propensity of *spinatus* muscles to form adipose tissue following RCT (Barry et al., 2013; Lee et al., 2020a). Hence, differences in the quantity and quality of PDGFRα+ FAPs at steady-state levels may reflect their heterogeneity and plasticity following damage. These findings are also consistent with a model where the variation in FAP content between muscle groups is explained by the same intrinsic and extrinsic determining factors that influence FAP behavior following chronic and persistent injury. Remarkably, two recent similar studies suggested that intramuscular cell transplantation of UCP1+ FAPs into a denervated RC tendon tear model enhanced vascularization, myofiber size, and mitigated atrophy of the RCT affected muscle, whereas reducing fatty deposition and myodegeneration (Lee et al., 2020b, c). The amelioration of massive RCT pathology by PDGFRα+ cell transplantation correlates with improved muscle quality and shoulder function after RC repair (Lee et al., 2020b, c). Thus, the first preclinical approaches using PDGFRα+ FAP cultures for muscle repair in rotator cuff tears suggest safety and potential efficacy, although the action mechanisms are still unclear.

In summary, these studies further demonstrate that PDGFRα+ FAPs are the primary cell source of the fibrous and fat tissues found in RCT disease. They also suggest the presence of PDGFRα+ cell subpopulations that favor either permanent scar formation or successful regeneration. These studies also suggest that fibro-adipogenic progenitors are promising candidates for cellular therapy to increase vascularization and insufficient repair of patients with advanced RCT disease or muscle ischemia.

## Altered Fate and Number of Muscle Fibro-Adipogenic Progenitors in Aging

**FIGURE 5:**
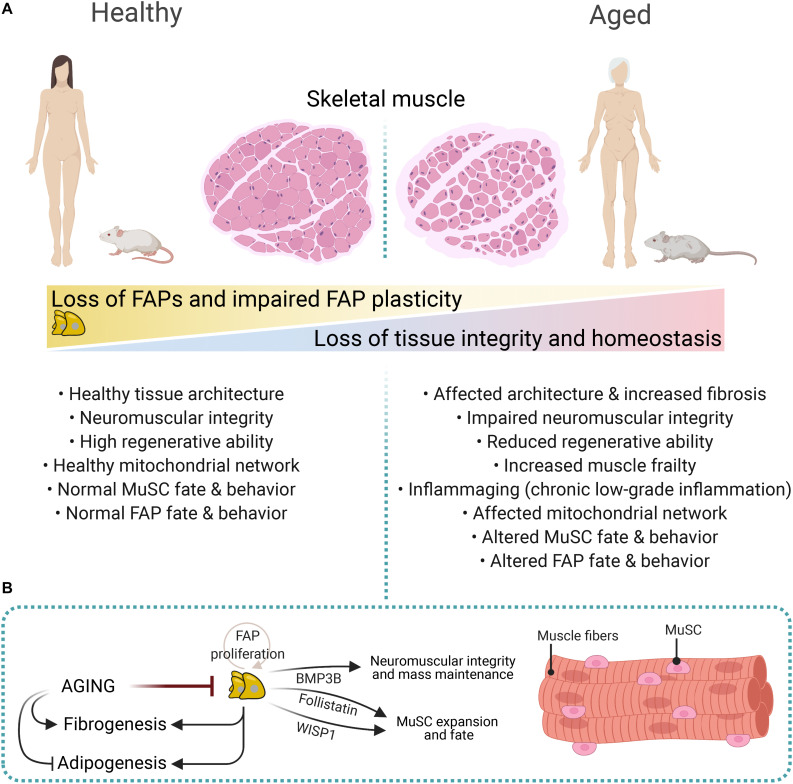
Impaired FAP numbers, behavior, and fate in aged muscles. **(A)** Sarcopenic and cachexic muscles have altered muscular integrity and structure, impaired neuromuscular communication, reduced regenerative capacities, enhanced fibrosis, and altered FAP number, fate, and behavior. These structural, cellular, and molecular age-related changes are associated with increased muscle frailty and muscle mass loss. **(B)** FAP-derived molecules regulate neuromuscular integrity, muscle mass, and MuSC expansion and fate. These include Follistatin, WISP1, and BMP3B. Aging impairs FAP proliferation and expansion, whereas it promotes fibrogenesis over adipogenesis.

The regenerative potential of muscles declines with age and associates with increased fibrosis and low-grade chronic inflammation (Figure 5A) (Brack et al., 2007; Brack and Rando, 2008; Etienne et al., 2020; Rahman et al., 2020). Impaired muscle stem cell fate and behavior, growth factors availability, altered stem cell niche, ECM composition and stiffness, loss of youthful systemic factors, and intrinsic molecular changes in muscle-resident cells are also associated with reduced muscle mass and increased atrophy, and tissue frailty with age (Figure 5A) (Etienne et al., 2020; Muñoz-Cánoves et al., 2020). Besides, aging primes FAPs to a fibrogenic state (Mueller et al., 2016; Lukjanenko et al., 2019). Recently, Lukjanenko et al. (2019) showed that aged FAPs have reduced proliferative capacity and adipogenic potential compared to young FAPs. Remarkably, the enhanced adipogenesis of cultured young *mdx*-FAPs is also reduced in aged mdx-FAPs (Mozzetta et al., 2013). Lukjanenko et al. (2019) also showed that the aberrant secretion of ECM-related molecules from PDGFRα+ cells during aging, particularly the reduced production of the matricellular protein CCN4/WISP1 (Cellular Communication Network Factor 4, WNT1-Inducible-Signaling Pathway Protein 1), disrupts MuSC behaviour and therefore, impairs muscle regeneration in old mice (Figure 5B). Notably, *in vivo* treatment of aged muscles subjected to acute injury with recombinant WISP1 increased the early proliferation of aged MuSCs and commitment of Pax7+/MyoD+ MuSCs, whereas it enhanced the proportion of newly formed myofibers and improved muscle architecture and myofiber cross-sectional area (Figure 5B) (Lukjanenko et al., 2019). Hence, systemic WISP1 administration ameliorates the impaired regenerative potential of aged muscles suggesting that FAP-secreted molecules could serve as potential therapeutics to boost the endogenous regenerative potential of muscles.

Aged muscles have reduced numbers of muscle-resident FAPs, and individual aged FAPs show decreased expression of PDGFRα compared to young counterparts (Figure 5B) (Uezumi et al., 2021). Reduced expression of PDGFRα in muscle and heart FAPs is also seen after damage (Contreras et al., 2019a; Soliman et al., 2020). Notably, FAP-derived Bone Morphogenetic Protein 3B (BMP3B, also known as Growth/Differentiation Factor-10, GDF10) has pleiotropic effects on muscle myofibers and Schwann Cells by stimulating hypertrophic signaling pathways and Schwann cell characteristics, which positively influences NMJ stability and muscle integrity (Uezumi et al., 2021). These results strongly suggest that aging-related changes influence FAPs’ homeostatic supportive role (Figure 5). Hence, the modulation of FAP-derived cues offers excellent therapeutic potential for combating muscle cachexia and sarcopenia.

Mozzetta et al. (2013), utilizing co-culture experiments between MuSCs and FAPs, showed that aging impairs the *mdx*-FAP-stimulated formation of MuSC-derived multinucleated myotubes. The authors also corroborated *in vivo* that the co-transplantation of FAPs improved the engraftment potential of MuSC as well as muscle regeneration into old mdx mice. The authors suggested that FAP-derived Follistatin mediates their pro-myogenic effects on MuSCs, which is enhanced by treatment with HDACi (Figure 5B) (Mozzetta et al., 2013). Mechanistically, the HDACi-mediated pro-regenerative muscle effects seem to involve a FAP-mediated release and transfer of miRNA-containing extracellular vesicles to support MuSCs and other cell types (Mozzetta et al., 2013; Sandonà et al., 2020). Taken together, these results suggest that despite the central role of muscle stem cells in mediating the regenerative potential of muscles and its loss during aging (Cosgrove et al., 2014; Sousa-Victor et al., 2015), muscle-resident FAPs also exert a significant influence on regenerative failure with aging. The extrinsic cues or intrinsic molecular determinants that affect these stromal progenitor cells as the body and muscle tissue age are unknown to date.

## Discussion

Normal muscle regeneration occurs in a complex and tightly regulated sequence of overlapping phases involving loss of homeostasis, inflammation, proliferation, tissue remodeling, and damage resolution. The connective tissue performs central functions at all these stages through its structural role as well as the matrix-building, remodeling, and secretory activities of its cells. Nevertheless, muscle pathology, disease, fibrosis, and aging compromise these coordinated responses and leads to progressive and permanent scarring and tissue failure. Over the past decades, significant efforts have been made to identify and characterize the cells responsible for forming the wound scar or interstitial fibrosis within muscles. The significant outcome has been the molecular and functional characterization of a population of PDGFRα expressing cells known as fibro-adipogenic progenitors. However, several outstanding questions remain open within the field (Box 2). For example, how are FAP phenotype and function regulated spatiotemporally as skeletal muscle chronic damage, muscle degeneration, and aging-related sarcopenia and cachexia progress? What role(s) do FAPs play in regulating the onset and progression of neuromuscular disease? How does the interaction of FAPs and nerve cells affect muscle, nerve, and tissue integrity? In homeostatic settings, how is the cross-talk between immune cells and FAPs regulated? Can muscle-resident FAPs or subpopulations of them be therapeutically targeted to slow or prevent muscle degeneration and fibro-fatty-bone infiltration? Addressing these should enhance our knowledge about the biology behind muscle FAPs and improve our current cell-based regenerative approaches and pharmacological treatments for debilitating muscle diseases and pathology.

**Box 2. Outstanding questions.**

(1) Do all FAPs originate from a common lineage during development? If not, which cell types can transition into FAPs?
(2) How are FAP phenotype and function spatiotemporally regulated during the progression of skeletal muscle chronic damage, muscle degeneration, and aging-related sarcopenia and cachexia?
(3) What is the role(s) of FAPs in regulating neuromuscular disease onset and progression? How does FAPs-nerve cell communication impact muscle, nerve, and tissue integrity?
(4) How is the cross-talk between immune cells and FAPs regulated in homeostatic conditions?
(5) Can the mapping of the molecular landscape of FAPs help us identify FAP-dependent pro-regenerative or pro-degenerative pathways?
(6) Can muscle-resident FAPs or sub-populations of them be targeted therapeutically to delay or affect muscle degeneration and fibro-fatty-bone infiltration?
(7) Can specific FAP sub-populations offer therapeutic windows in pathology and disease?

PDGFRα+ fibro-adipogenic progenitors are essential mediators of successful skeletal muscle regeneration and participate in tissue homeostasis and integrity. A growing body of evidence supports the hypothesis that the *in vivo* expansion and differentiation of FAPs mostly depends on the tissue environment, and thus, is extrinsically dictated by the surrounding niche. The notions of PDGFRα+ FAP cells with intrinsic pro-regenerative, pro-inflammatory, and pro-fibrotic roles parallel their cell heterogeneity and fate. Nevertheless, their dysregulated activity directly leads to tissue scarring in muscle ischemia, pathology, neuromuscular disorders, and aging. Understanding FAP physiology will lead to significant advances in comprehending the pathogenesis of muscle fibrosis and exploring new therapeutic options for treating debilitating diseases in which permanent scars tip the balance to tissue malfunctioning and organ failure.

## Author Contributions

MT and OC conceived, designed, and drafted the manuscript and figures. FR edited and reviewed the manuscript. All authors read and approved the final version of the manuscript prior to submission.

## Conflict of Interest

The authors declare that the research was conducted in the absence of any commercial or financial relationships that could be construed as a potential conflict of interest.

## Abbreviations

AMPK: AMP-activated kinase
BMP2: bone morphogenetic protein 2
BMP9: bone morphogenetic protein 9
CFU: colony-forming unit
CT: connective tissue
DAMP: damage-associated molecular pattern
DMD: duchenne muscular dystrophy
ECM: extracellular matrix
FSHD: facioscapulohumeral dystrophy
FAPs: fibro-adipogenic progenitors
FOP: fibrodysplasia ossificans progressive
HO: heterotopic ossification
HIF-1a: hypoxia-inducible factor 1a
IL: interleukin
IMAT: intermuscular adipose tissue
LGMD: limb-girdle muscular dystrophy
Ly6C: lymphocyte antigen 6 complex
MPs: macrophages
MSCs: mesenchymal stem cells
MCT: muscle connective tissue
MuSCs: muscle stem cells
PDGFRα: platelet-derived growth factor receptor-alpha
PDGFRβ: platelet-derived growth factor receptor-beta
PDGF: platelet-derived growth factor
PD-1: programmed death-1
PD-L1: programmed death-Ligand 1
SMA: spinal muscular atrophy
SCA-1: stem cell antigen-1
TGF-β: transforming growth factor type beta
TNF-α: tumor necrosis factor-alpha.
